# Bioaccessibility and Oxidative Stability of Omega-3 Fatty Acids in Supplements, Sardines and Enriched Eggs Studied Using a Static In Vitro Gastrointestinal Model

**DOI:** 10.3390/molecules27020415

**Published:** 2022-01-09

**Authors:** Stylianos Floros, Alexandros Toskas, Evagelia Pasidi, Patroklos Vareltzis

**Affiliations:** 1 Department of Chemical Engineering, Faculty of Engineering, Aristotle University of Thessaloniki, 54124 Thessaloniki, Greece; florstyl@cheng.auth.gr (S.F.); egpasidi@cheng.auth.gr (E.P.); 2 Petros Androulakis Medical Biology Analytical Laboratories, 57001 Thermi, Greece; alex_tsks@hotmail.com

**Keywords:** omega-3 fatty acids, bioaccessibility, in vitro digestion, oxidation, functional foods, supplements

## Abstract

Modern dietary habits have created the need for the design and production of functional foods enriched in bioactive compounds for a healthy lifestyle. However, the fate of many of these bioactive compounds in the human gastrointestinal (GI) tract has not been thoroughly investigated. Thus, in the present study, the bioaccessibility of omega-3 fatty acids was examined. To that end, different foods and supplements underwent simulated digestion following the INFOGEST protocol. The selected samples were foods rich in omega-3 fatty acids both in free and bound form—i.e., dietary fish oil supplements, heat-treated fish, and eggs enriched with omega-3 fatty acids. The oxidation of polyunsaturated fatty acids (PUFAs) was measured at each stage of the digestion process using peroxide value (PV) and TBARS and by quantifying individual omega-3 fatty acids using a gas chromatograph with flame ionization detector (GC-FID). The final bioaccessibility values of omega-3 fatty acids were determined. Changes in the quantity of mono-saturated fatty acids (MUFAs) and saturated fatty acids (SFAs) were recorded as well. The results indicated a profound oxidation of omega-3 fatty acids, giving rise to both primary and secondary oxidation products. Additionally, stomach conditions seemed to exert the most significant effect on the oxidation of PUFAs during digestion, significantly decreasing their bioaccessibility. The oxidation rate of each fatty acid was found to be strongly correlated with its initial concentration. Finally, the oxidation pattern was found to be different for each matrix and emulsified lipids seemed to be better protected than non-emulsified lipids. It is concluded that digestion has a profound negative effect on omega-3 bioaccessibility and therefore there is a need for improved protective mechanisms.

## 1. Introduction

Omega-3 fatty acids are polyunsaturated fatty acids (PUFAs) that are characterized by the presence of multiple double bonds, where the first one is located three atoms away from the terminal methyl group in their chemical structure [1]. Certain omega-3 fatty acids are essential elements of the human diet because they have numerous effects on health based on their function and nutritional value and cannot be synthesized biologically through the human metabolic processes [1,2]. Some well-known omega-3 fatty acids, such as C18:3 (a-linoleic acid), C20:5 (eicosapentaenoic acid), and C22:6 (docosahexaenoic acid), play multiple roles in the human body [2]. The omega-3 fatty acids prevent heart diseases, have an anti-inflammatory role, resist autoimmune diseases, maintain homoeostasis, and support brain function via their essential presence in signal transmission and many more functions [2].

At the same time, consumers’ awareness of food-related health issues is rising and leading to a higher consumption of omega-3 fatty acids due to the health benefits attributed to PUFAs. Industries and researchers have focused on tackling problems related to the management of these components. PUFAs are prone to oxidation during isolation, processing, and storage and are very sensitive to the conditions in the human gastrointestinal (GI) tract [3,4,5,6]. The oxidation of omega-3 fatty acids leads to the production of potentially harmful primary and secondary oxidation products [3,4], where, in addition to causing lipid malabsorption and ultimately lipids deficiency [7], they also threaten the health of the human organism if consumed over a long period of time [2,3,4,8].

There is a controversy in the literature regarding omega-3 bioavailability, while the interactions of individual fatty acids with the human digestive tract are not yet fully elucidated [6]. Therefore, it is necessary to investigate their bioaccessibility as they pass through the human gastrointestinal tract. By determining the bioaccessibility of omega-3 fatty acids, important information that will contribute to the management, modification, and enhancement of a food’s or supplement’s final bioaccessibility and subsequent bioavailability can be derived [9]. To extract information about bioaccessibility, it is necessary to simulate the effects of the human gastrointestinal system using an in vitro digestion protocol [10,11]. Recently, the INFOGEST in vitro static/dynamic protocol was proposed. It has since been widely used, as it is described as one of the simplest, most accurate, and most reproducible protocols available [10].

Free fatty acids (FAs), including the omega-3 ones, when found either isolated or in a food matrix can combine with other lipid compounds—i.e., monoglycerides (MAGs) and phospholipids—to form compounds widely known as triglycerides (TAGs) [4,5,6,7,12]. During their passage through the gastrointestinal tract, triglycerides undergo enzymatic and chemical reactions and are transported from the intestine to peripheral tissues in chylomicrons or very low density lipoproteins (VLDL), or both [13,14,15]. This transformation process is mainly characterized by emulsification and enzymatic hydrolysis, which are carried out by gastric and intestinal salts and lipases [15]. According to the literature, triglycerides undergo a two-step reaction, where one mole of triglyceride produces one mole of fatty acid and one mole of 1,2-diglyceride. This step is followed by hydrolysis, finally leading to the production of one mole of 2-monoglyceride and two moles of fatty acids. In most of these cases, further oxidation occurs, transforming omega-3 fatty acids into degraded oxidation products. Therefore, omega-3 fatty acids are unavoidably exposed to the pro-oxidant conditions of the gastrointestinal tract [12,13]. Tullberg C. et al. investigated different types of fish oils and found that there was a rise in oxidation products after digestion [16]. Larrson K. et al. showed that the presence of anti-oxidants in fish oil was not enough to significantly retard lipid oxidation throughout the GI tract [17].

The aim of this study was to investigate the effect of the digestive tract on the oxidative stability and bioaccessibility of omega-3 fatty acids. This was achieved by implementing the INFOGEST protocol to three categories of samples: (a) dietary supplements rich in omega-3 fatty acids (four different brands of chewable tablets and capsules); (b) sardines, as an example of a natural source of omega-3 fatty acids; and (c) omega-3 enriched eggs, as an example of a fortified food. The effect of heat treatment on the oxidation of PUFA from natural dietary sources prior to digestion was also determined. The derived information can be utilized to enhance the final bioaccessibility of the omega-3 fatty acids and benefit consumers’ health via the proper modification of dietary products.

## 2. Results

Fish oil supplements, sardines, and eggs enriched with omega-3 FA were exposed to simulated gastrointestinal conditions (INFOGEST protocol), and the oxidation of omega-3 was followed at each stage by PV, TBARs, and GC-FID. Since fish and eggs are usually cooked before consumption, these samples were also heat-treated at 70 °C for 20 min prior to simulated digestion.

### 2.1. Primary Oxidation—PV Determination

Multiple independent experiments were performed to investigate the effect of the GI tract’s conditions on the oxidation stability of four different brands of commercially available fish oil supplements (A, B, C, D), sardines, and eggs enriched with omega-3 fatty acids. The results are summarized in Table 1. It is observed that PV is significantly increased by the end of the simulated digestion for all samples. Among the supplements, the highest increase (615%) was observed for Brand A, which consisted of concentrated fish oil containing a small amount of antioxidant vitamins, which were unable to retard lipid oxidation and protect the omega-3 fatty acids throughout digestion. Brand C had the highest initial and final PV in absolute numbers among supplements, but the smallest increase (245%). It can also be seen that the higher the initial PV was, the higher the corresponding value at the end of the digestion was, which can be explained by the fact that peroxides are potent pro-oxidants and can lead to the generation of more peroxides via the autooxidation mechanism. The differences in oxidative stability among the supplements can be attributed to their different composition (type and concentration of antioxidants in their formulation) and physical state (solid or liquid form). Regarding food samples, it was observed that omega-3 enriched eggs started with a significantly higher peroxide value compared to sardines, but the increase was only close to 100%, while for sardines the increase was over 600%. The heat treatment of both food samples did not seem to significantly affect their oxidative stability (*p* > 0.05).

**Figure 1:**
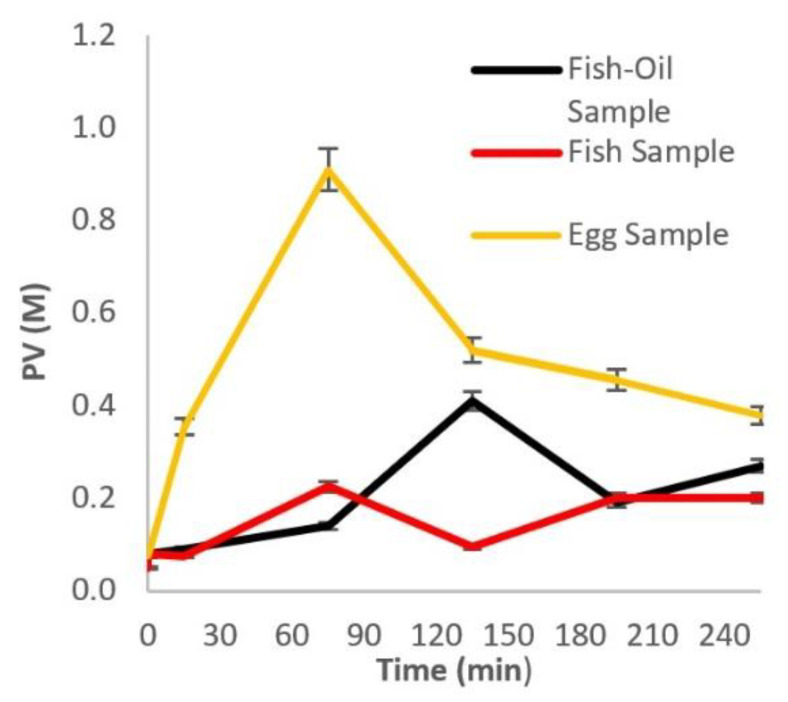
Effect of the GI tract on the peroxide production of fish oil supplement: Brand D; sardine; and fortified egg.

To gain a deeper understanding of the oxidation process during digestion, samples were taken and analyzed at each step of the digestion. The results in Figure 1 for the supplement Brand D show that there was a lag phase up to 80 min, probably due to the protection of the antioxidants present in the supplements’ formulation and/or the protective layer surrounding the omega-3s, followed by a sharp increase in the concentration of peroxides during the gastric stage (stomach). This indicates the profound influence of this stage on the oxidation of omega-3 FA. The following sharp decrease in PV can be attributed to the nature of peroxides, which are very unstable compounds and can either participate in other reactions or give rise to secondary oxidation products through the b-scission mechanism [18,19]. Heat-treated sardine samples exhibit a two-stage oxidation behavior (Figure 1). The results in Figure 1 also show that the pro-oxidative gastric conditions play the most important role in the oxidation of the heat-treated omega-3-enriched eggs.

### 2.2. Secondary Oxidation—TBARS Determination

The excess lipid peroxidation due to the pro-oxidative nature of the human GI tract has led to the production of secondary oxidation products. These compounds have been linked to pathophysiological problems, and the chronic exposure of the human body to their consumption may result in organic damage and abnormalities [20]. Secondary oxidation was followed by determining the malondialdehyde equivalents (MDA_eq_) using the TBARS method. These MDA_eq_ are generated throughout the passage of the dietary lipids along the GI tract and consist of an important indicator of quality degradation and of the general oxidation of polyunsaturated fatty acids [4].

Table 2 lists the results of the TBARS test in four different brands of omega-3 FA dietary supplements and fish and egg samples. Regarding the dietary fish oil supplements, there appears to be a significant increase (*p* > 0.05) in secondary oxidation products at the end of the passage throughout the GI tract, regardless of their initial composition and the presence of antioxidants and/or coating material. The heat treatment of both sardines and eggs did not seem to significantly affect their MDA values (*p* > 0.05). Similarly to the supplements, a significant increase in MDA was recorded for sardines and eggs by the end of digestion. However, the % increase in these foods was significantly lower compared to that of the supplements, indicating the possible role of the natural antioxidant mechanisms and/or matrix.

**Figure 2:**
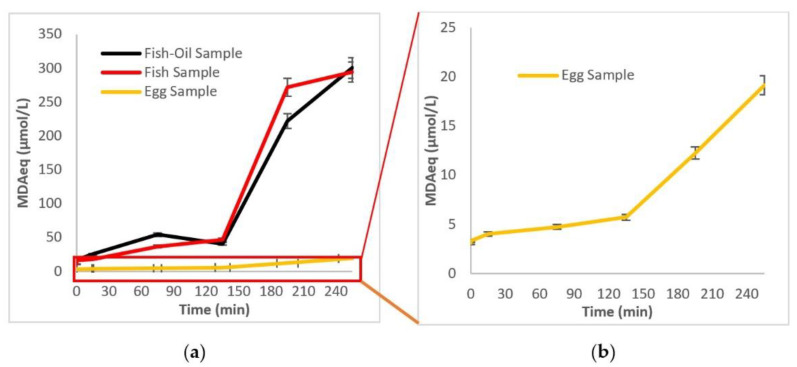
Effect of the GI tract on secondary oxidation of (**a**) fish-oil supplement—Brand D; sardine; fortified egg (**b**) close view—(zoom) on the egg sample.2.

To further investigate the evolution of secondary oxidation products during the digestion process, more frequent sampling and MDA_eq_ determination were performed, as shown in Figure 2. The results show that for all samples (supplement Brand D, heat-treated sardines, and eggs) the increase in MDA_eq_ was profound after the first 120 min of digestion. Since secondary oxidation products are mainly produced by the decomposition of pre-existing and/or newly formed peroxides, it was expected that the MDA_eq_ would increase at a later time than PVs. This coincided with the beginning of the intestinal phase of digestion for all samples. Secondary oxidation seems to be milder for the egg samples compared to the other samples, as shown in Figure 2a,b.

The results of the PV and TBARS suggest a high degree of oxidation of polyunsaturated fatty acids (PUFA) in all samples as they pass through the human GI tract. However, the calculation of omega-3 FA bioaccessibility requires a more detailed analysis. Gas chromatography was employed to calculate the bioaccessibility index of the individual FAs and of the total omega-3 by quantifying the exact amount of omega-3 FA. The initial and final (after digestion) concentrations of individual omega-3 FAs of the samples (four supplements, fish, and eggs) and their corresponding bioaccessibility index (BI) are summarized in Table 3.

The calculated BI represents the ability of an independent omega-3 FA to resist the oxidative environment of the GI tract, come to a bioavailable state, and be transported to the lymph system. The results also show an increase and/or decrease in the concentration of certain saturated (SFA) and monounsaturated fatty acids (MUFA) (Table S1 of the Supplementary Materials contains the list of all the fatty acids and their nomenclature, while Table S2 shows their concentrations). Specifically, the change in SFA is justified due to the strong oxidative action of the environment of the GI tract, as well as the production of oxidation products. Thus, the transition of FA from PUFA to MUFA and SFA, and the increase or reduction in the SFA during oxidation, leading to much smaller oxidation products, is noticeable [21].

It is noteworthy that three out of four supplements showed a poor BI score (30–35%), indicative of their susceptibility to lipid oxidation during digestion. Only supplement Brand B retained most of its PUFAs intact, probably due to its composition and/or physical state (emulsified lipids in solid instead of liquid state). It was also noted that PUFAs in fish samples had a significantly higher BI than the egg PUFAs.

**Figure 3:**
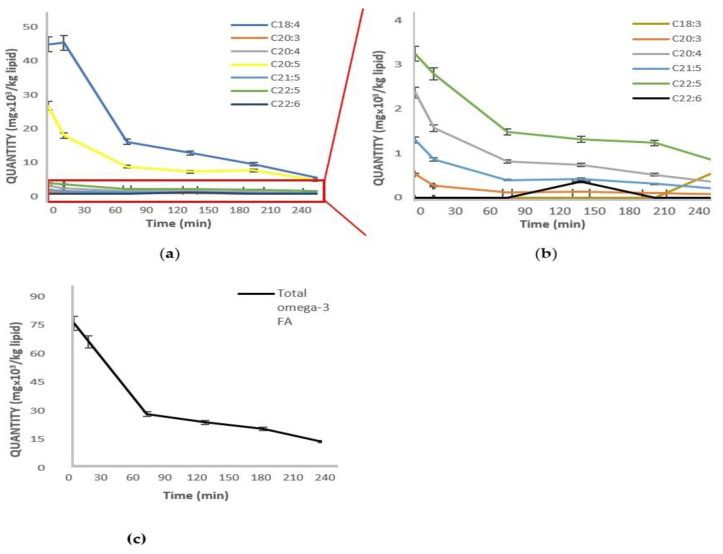
(**a**) Effect of the GI tract on the oxidation of individual omega-3 FAs of fish oil supplement: Brand D; (**b**) close view (zoom) of some omega-3 FA from a; (**c**) effect of the GI tract on the oxidation of total omega-3 FAs.

To gain a better insight into the progression of the oxidation of individual PUFAs during digestion, samples of supplement Brand D were taken from different stages of the simulated process and analyzed by GC-FID (Figure 3a,b). It was concluded that, overall, the highest rate of oxidation took place in the first 75 min of digestion (gastric phase) (Figure 3c). This is in accordance with the results from PV and TBARS, as shown in the previous sections. It was also observed that the higher the initial concentration of a specific PUFA was (e.g., C18:4, C20:5), the higher the degree of oxidation was (Figure 3a). This was verified by calculating the average initial rate of oxidation of the individual PUFAs (Table 4). There was a strong correlation (r = −0.9993) between the initial concentration and oxidation rate, regardless of the length of the chain and degree of unsaturation.

## 3. Discussion

PUFAs, especially omega-3 fatty acids, have been the center of attention in both the industrial and scientific community in recent years due to their beneficial attributes for human health. However, due to their nature, these fatty acids are prone to oxidation and quality deterioration. After consumption, they are interacting with the pro-oxidative environment of the digestive tract, often leading to limited bioavailability. These interactions involve different dynamic physicochemical and physiological processes. These processes depend on the structural and physicochemical properties of the initial food, as well as the characteristics of the consumer (e.g., age, sex, genetics, health, etc.) and various other factors (time of consumption, previous food consumed, temperature) [13]. Various techniques have been developed in order to protect these lipids from oxidation when consumed either as supplements or when processed as part of foodstuffs—i.e., encapsulation, the addition of antioxidants, and emulsification [22]. However, there is still the need to clarify the interaction of these components with the environment of the GI tract. Researchers have come to realize that several nutrients do not behave in a similar manner when studied isolated than in whole foods. Matching chemical composition does not guarantee similar behavior in nutrient delivery and biological function [23].

In this study, different matrices of dietary omega-3 were chosen as samples to investigate the behavior of PUFAs during digestion. Four different dietary supplements (Brand A in the form of a soft capsule containing concentrated fish oil and antioxidants, Brand B in the form of a soft chewable capsule containing an emulsion of concentrated fish oil and antioxidants, Brand C in the form of a soft capsule containing concentrated fish oil and seed oil, and Brand D in the form of a soft capsule containing concentrated fish oil and antioxidants) were tested. Sardines were chosen as a representative natural source of PUFAs and omega-3 fortified eggs as an example of a functional food. Heat treatment of these natural sources of PUFAs did not affect their oxidation status. Similar observations were made by Leung et al., where the effect of the use of different cooking methods on fish lipid peroxidation was investigated [24].

This study shows that, regardless of the raw material used, there was a high degree of oxidation by the end of the digestion process, as indicated by the PV, TBARS, and individual fatty acid concentrations. This leads to the decreased bioaccessibility of omega-3 fatty acids, as indicated by the calculated BI. However, oxidation generates various potentially bioavailable products that can be found in the sample at the end of digestion. The effect of oxidized omega-3 fatty acids on consumers’ health is still under investigation in the literature. According to several researchers, oxidative products might be linked to cardiovascular issues and/or cancer. It is postulated that the consumption of oxidized products will lead to the inflammation and oxidation of tissues in the human body. However, this is still a controversial issue. Oxygenated lipids possess a physiological signaling role and it has not yet been clarified whether the oxidized lipid products are effectively absorbed or whether their role is comparable to that of oxygenated lipids [25,26]. Further experiments and clinical trials need to be conducted in order for this controversy to be resolved.

Oxidation at each step of the digestion process revealed that not all samples followed the same pattern (Figure 1 and Figure 2). Supplement D exhibited a significant lag phase regarding peroxides and TBARS, probably due to the presence of antioxidants and coating material. Supplement Brand B also had a higher BI compared to the other supplements, probably due to its emulsified physical state. The effect of the digestion enzymes (pepsin, gastric lipase, pancreatic lipase, pancreatin, bile bovine) on food molecules has been thoroughly described in the literature. It has been reported in some cases that the enzymes are unable to break down the incoming structure, as is often the case with emulsion matrices, due to surface stresses and stereochemical inhibitions. The same phenomenon can affect bioavailability due to the inadequacy of the raw material that needs to be emulsified or incorporated into structures such as micelles and become bioavailable [27,28]. Another important observation was that sardines followed a two-stage oxidation according to PV measurements. This can be attributed to the gradual release of PUFAs from the matrix caused by acid hydrolysis during the gastric phase and the action of lipophilic enzymes in the intestinal phase [29]. Secondary oxidation pattern and BI were comparable to that of three supplements. This can be attributed partly to the intrinsic antioxidant mechanisms present in the muscle tissue of the fish. Among many enzymes, the most important type of enzyme-catalyzing reduction of hydroperoxides is glutathione peroxidase (GPx), where lipid hydroperoxides are reduced in a reaction, leaving lipid hydroxide, oxidized glutathione, and selenocysteine residue [30]. The oxidation in eggs was also profound during the gastric phase, followed by a slower decrease in the intestinal phase for the peroxides, while TBARS showed less oxidation compared to the other samples, especially the supplements. This can be attributed to the release of antioxidant peptides during digestion. Studies have shown that the release of amino acids and antioxidant peptides during egg digestion leads to the overall increase in their anti-oxidative capacity while maintaining the bioaccessibility of the antioxidants zeaxanthin and lutein, which naturally occur in egg [31,32,33,34]. Nolasco et al. reported that linoleic acid was not detected after simulated digestion, but other PUFAs in enriched eggs did retain their bioaccessibility [35]. The source of PUFAs in chicken feed, their form and concentration, physiological characteristics (age, size, etc.) and other factors can affect the final bioaccessibility and bioavailability [36].

Comparing Figure 1 and Figure 2, it can be seen that the pro-oxidative conditions of the stomach (gastric phase) lead to primary oxidation, while secondary oxidation mainly takes place in the intestinal phase. Therefore, it is imperative to protect the PUFAs during the gastric phase to limit the production of primary oxidation products. For instance, it was shown by Venugopalan V.K. et al. that emulsifying PUFAs into small lipid droplets can enhance their bioavailability [37]. Additionally, according to Chang H.W et al., adding a coating through encapsulation can protect emulsified fish oils from degradation by gastric enzymes, leading to a more sustained release under intestinal conditions [38]. The ionic nature and the wall layer thickness of the coating seem to play a key role in maintaining the integrity of the emulsified oils. From the different food matrices and compositions tested in this study, only the supplement in the form of emulsion showed an adequate resistance to lipid oxidation during digestion. Antioxidants (either natural or added) on their own were not sufficient to protect PUFAs from the pro-oxidative conditions in the GI tract.

The rate and degree of oxidation depend on many factors, including the initial concentration of fatty acids, degree of unsaturation, physical state, presence of antioxidants, etc. In this study, the rate of oxidation of each individual PUFA in the case of supplement Brand D was found to be strongly correlated with its initial concentration. Several researchers have shown that concentration plays an important role in the development of lipid oxidation. Waraho et al. mentioned that the oxidative stability of emulsions is linked to both the concentration and type of the free fatty acid content, while Fereidoon S. et al. showed that phospholipids with a higher percentage of PUFAs exhibited greater oxidation rates [39,40].

Based on these results, we conclude that there is a severe oxidation of omega-3 FAs in dietary supplements, in natural sources (sardine), and even in functional foods (omega-3-fortified eggs). Primary oxidation products were predominant in the gastric stage, while secondary oxidation products were predominant in the intestinal phase of the digestion. This raises the question of the possible effects on human health that the oxidation products reaching the intestine might have. Most of the calculated BIs were considered to be very low to low depending on the nature of the sample. The results of this study provide valuable information that will contribute to the construction and optimization of delivery systems to protect omega-3 FAs and increase their bioaccessibility and bioavailability.

## 4. Materials and Methods

### 4.1. Chemicals and Reagents

Bile bovine dried, cumene hydroperoxide, *p*-toluene-sulfonyl-L-arginine methyl ester (TAME), glyceryl tributyrate (tributyrin), sodium taurodeoxycholate, Tris-(hydroxymethyl)-aminomethane (Tris), tetraethoxypropane (TEP), potassium chloride (KCl), sodium bicarbonate (NaHCO_3_), calcium chloride (CaCl_2_), and magnesium chloride (MgCl_2_) were purchased from Merck & Co. (New Jersey, NJ, USA). Sodium chloride (NaCl), potassium dihydrogen phosphate (KH_2_PO_4_), hydrochloric acid 37% (HCl), chloroform (CHCl_3_), methanol (CH_3_OH), ammonium thiocyanate (NH_4_SCN), and iron (III) chloride (FeCl_3_) were purchased from Chem-Lab NV (Zedelgem, Belgium). Porcine pepsin, pancreatin, and ammonium carbonate (NH_4_)_2_CO_3_ were purchased from Central Drug House Ltd. (New Delhi, India). Butylated hydroxytoluene (BHT) and -thiobarbituric acid (TCA) were purchased from Fluorochem Ltd. (Hadfield, UK). Sodium hydroxide (NaOH) and trichloroacetic acid (TCA) were purchased from Lach-Ner Ltd. (Neratovice, Czech Republic). All the chemicals and reagents used in this study were of analytical or HPLC grade. The samples tested were various well-known brands of fish oil chewable tablets and capsules purchased from local pharmacies; fresh sardines that were purchased from local vendor; and eggs rich in omega-3 fatty acids, which were kindly offered by Avgodiatrofiki S.A. (Nea Santa, Kilkis, Greece).

### 4.2. Sample Preparation

#### 4.2.1. Enzyme Activity Assays

The oxidative behavior of PUFAs along the GI tract was studied by implementing of the INFOGEST protocol. The INFOGEST protocol is divided into 3 stages, starting with the preparation of the samples, which includes the characterization of the activities of enzymes and bile salts used. The full protocol stages are reported later. The activities of all enzymes purchased are given, except for pancreatin, which was calculated according to the pancreatin assay of the INFOGEST protocol. The characterization of pancreatin activity was normalized to the trypsin and pancreatic lipase activity assays [10].

#### 4.2.2. Stock Solution Preparation

According to the INFOGEST protocol, digestion involves the exposure of food to three phases (oral, gastric, and intestinal phase). The electrolytes used for every stage were prepared in advance in stock solutions and stored at –10 °C. Specifically, stock solutions of KCl (0.5 M), KH_2_PO_4_ (0.5 M), NaHCO_3_ (1 M), NaCl (2 M), MgCl_2_(H_2_O)_6_ (0.15 M), (NH_4_)_2_CO_3_ (0.5 M), HCl (0.09 M), and CaCl_2_(H_2_O)_2_ (0.025 M) were prepared. These stock solutions were used to create the simulated fluids (1.25×) for each stage of digestion, known as simulated salivary fluid (SSF), simulated gastric fluid (SGF), and simulated intestinal phase (SIF), as described sufficiently in the INFOGEST protocol manuscript [10].

### 4.3. Static In Vitro Simulation of Gastrointestinal (GI) Digestion

#### 4.3.1. Oral Digestion Phase

The protocol began with the preparation of the samples and their primary homogenization. One gram of food sample was added to a test tube and mixed with SSF (1.25×). Distilled water was added to achieve a final volume ratio of 1:1. The final mixture was transferred to a heated incubator, where the test tube was shaken under heating for 2 min at a constant temperature of 37 °C [10].

#### 4.3.2. Gastric Digestion Phase

The oral bolus was mixed with SGF (1.25×). Additionally, pepsin was solubilized with water to reach a final activity of 2000 U/mL and added to the mixture. The pH was set to 3 by the addition of the HCl solution (1 M). Distilled water was added until a final volume ratio of 1:1 was reached. The final mixture was transferred to the temperature-controlled incubator, where it remained for 2 h at a temperature of 37 °C [10].

#### 4.3.3. Intestinal Digestion Phase

The gastric chyme was mixed with SIF (1.25×), pancreatin solution (100 TAME U/mL), and bile salt solution (10 mmol/L). The pH was set to 7 by the addition of NaOH solution (1 M). Distilled water was added until a final volume ratio of 1:1 was reached. The mixture was finally transferred to the temperature-controlled incubator, where remained for 2 h at a constant temperature of 37 °C [10].

### 4.4. Sample Treatment and Storage

BHT (500 ppm) was added after the end of the protocol to inhibit further oxidation, while samples were frozen and kept at −20 °C until further evaluation [41].

### 4.5. Sample Analysis

#### 4.5.1. Peroxide Value (PV)

The PV was measured according to a modified method as described by Richards et al. [42]. One gram of the lipid sample was mixed with 10 mL of CHCl_3_-CH_3_OH (2:1, *v*/*v*). Then, 500 ppm of BHT was added to the test tube to stop the oxidation process. The mixture was homogenized for 15 s and then filtered to remove solids. Then, 1.5 mL of NaCl (0.5%) was added and the mixture was vortexed and centrifuged at 4.000 rpm for 10 min to separate the 2 phases at ambient temperature. After centrifugation, the lower phase of CHCl_3_ was collected and a quantity of CHCl_3_-CH_3_OH (2:1) was added until a final volume of 10 mL was reached. Lastly, 25 μL of NH_4_SCN solution (30% *w*/*v*) and 25 μL of FeCL_3_ solution (0.66% *w*/*v*) were added, and the mixture was vortexed for 2–4 s. Peroxides were measured by spectroscopic absorption at 500 nm using a Quartz cell. As a blind sample, 10 mL of 2:1 CHCl_3_-CH_3_OH mixture was used; the oxidation products were expressed in mg/L of lipid phase using a standard curve formed with cumene hydroperoxide solutions [43,44,45].

#### 4.5.2. Thiobarbituric Acid (TBARS) Method

The TBARS were determined according to Lemon with small modifications. Briefly, an initial amount of 1.5 g of the sample was added to a test tube with 5 mL of TCA (7.5% *w*/*v*). The mixture was homogenized, vortexed, and centrifuged for 25 min at 4.000 rpm. An aliquot of 2 mL was mixed with 2 mL of TBA solution (0.02 M). The mixture was heated in a water bath for 40 min at a constant temperature of 100 °C. Finally, the absorbance was measured spectroscopically at 532 nm. As a blind sample, TBA: TCA solution (1:1) was used and the oxidation products were expressed as MDA_eq_ (ppm) with the help of a standard curve constructed using TEP solutions [46].

#### 4.5.3. Gas Chromatography—Flame Ionization Detector (GC-FID)

Each sample was homogenized and total lipids were extracted using the Folch method [47]. Specifically, 1 g of each homogenized sample was mixed with 20 mL of CHCl_3_-CH_3_OH (2:1 *v*/*v*). The mixture was vortexed and water was added for the phase separation. The upper phase was removed and the lower one was collected. After solvent evaporation under a dry nitrogen atmosphere, 0.1 g of the extracted lipids were weighted in a test tube with a screw cap. Afterwards, the samples were reconstituted in 2 mL of hexane, followed by the addition of 0.2 mL of a 2 M methanolic solution of KOH for the preparation of fatty acid methyl esters (FAMEs). The mixture was vortexed for 1 min and left to settle till the upper phase became transparent. Then, the upper phase containing the methyl esters was collected and analyzed by gas chromatography with a flame ionization detector (Agilent 8890 GC System) equipped with an autosampler (Agilent 7693A System). FAMEs were analyzed on a HP-88 column (100 m × 0.25 mm; 0.20 μm, J &W Scientific, Agilent Technologies, Santa Clara, CA, USA). Hydrogen was the carrier gas at a flow rate of 0.928 mL/min. The injector port and detector temperature were maintained at 260 °C. The column (HP-88) oven was programmed to maintain a temperature of 100 °C for 5 min, which then rose to 180 °C at 8 °C/min, where it was maintained for 9 min, before rising to a plateau of 230 °C at a rate of 1 °C/min for 15 min. The total run time was 89 min. FAMEs were identified by comparing retention times to a standard mixture containing 37 fatty acids and the PUFA standard No. 3 from Merck containing unsaturated fatty acids.

### 4.6. Bioaccessibility Index (BI)

To estimate the bioaccessibility of omega-3 fatty acids during gastrointestinal digestion, the bioaccessibility index (BI) was determined using the following equation:

BI (%) = (C_b_/C_a_) × 100) (1)

where C_a_ and C_b_ are the amounts of each PUFA before and after digestion [48]. BI was calculated for PUFAs that were present in the sample before digestion (C_a_ > 0).

### 4.7. Statistical Analysis

At least three independent digestion experiments were conducted (*n* = 3) and the experimental results were expressed as the mean ± SD unless otherwise noted. Differences were considered significant at *p* < 0.05. A one-way ANOVA with Tukey’s test for comparison was carried out by the Minitab 21 Statistical Software (Minitab LLC, State College, PA, USA).

## Figures and Tables

**Table 1:** Peroxide value of samples before and after digestion.

Peroxide Concentration (M)				
Sample	Raw State (Before GI Tract)	Heat Treated State (Before GI Tract)	Digested State (After GI Tract)	Increase (%)
Fish Oil Supplement: Brand A (*n* = 3)	0.013 ± 0.006 ^a^	N/A	0.093 ± 0.045 ^b^	615
Fish Oil Supplement: Brand B (*n* = 3)	0.026 ± 0.011 ^a^	N/A	0.140 ± 0.026 ^b^	438
Fish Oil Supplement: Brand C (*n* = 3)	0.056 ± 0.011 ^a^	N/A	0.193 ± 0.025 ^b^	245
Fish Oil Supplement: Brand D (*n* = 3)	0.083 ± 0.005 ^a^	N/A	0.273 ±0.015 ^b^	229
Sardine (*n* = 9)	0.026 ± 0.005 ^a^	0.035 ± 0.006 ^a^	0.190 ± 0.010 ^b^	630
Egg (*n* = 9)	0.141 ± 0.027 ^a^	0.144 ± 0.053 ^a^	0.280 ± 0.077 ^b^	98

Table values are means ± standard deviations. Different superscript letters in the same row represent statistical differences (*p* ≤ 0.05); *n* represents the number of independent experimental replications.

**Table 2:** TBARS results before and after digestion.

MDA_eq_ Concentration (M × 10^−6^)				
Sample	Raw State (Before GI Tract)	Heat Treated State (Before GI Tract)	Digested State (After GI Tract)	Increase (%)
Fish Oil Supplement: Brand A (*n* = 3)	13.46 ± 2.92 ^a^	N/A	189.72 ± 99.84 ^b^	1310
Fish Oil Supplement: Brand B (*n* = 3)	7.14 ± 0.69 ^a^	N/A	147.65 ± 17.49 ^b^	1968
Fish Oil Supplement: Brand C (*n* = 3)	15.80 ± 9.76 ^a^	N/A	385.14 ± 36.50 ^b^	2338
Fish Oil Supplement: Brand D (*n* = 5)	13.05 ± 3.60 ^a^	N/A	232.29 ± 52.70 ^b^	1680
Sardine (*n* = 9)	64.09 ± 3.71 ^a^	48.27 ± 8.80 ^a^	196.99 ± 37.02 ^b^	207
Egg (*n* = 9)	6.13 ± 1.32 ^a^	6.17 ± 1.37 ^a^	19.75 ± 5.68 ^b^	222

Table values are means ± standard deviations. Different superscript letters in the same row represent statistical differences (*p* ≤ 0.05); *n* represents the number of independent experiment replications.

**Table 3:** Results of GC-FID and BI calculations.

**Fish Oil Supplement, Brand A**				**Fish Oil Supplement, Brand B**				**Fish Oil Supplement, Brand C**			
**PUFA**	**C_a_ (mg/kg** **Lipid)**	**C_b_ (mg/kg** **Lipid)**	**BI** **(%)**	**PUFA**	**C_a_ (mg/kg** **Lipid)**	**C_b_ (mg/kg** **Lipid)**	**BI** **(%)**	**PUFA**	**C_a_ (mg/kg** **Lipid)**	**C_b_ (mg/kg** **Lipid)**	**BI** **(%)**
C22:6 *n*-3 (DHA)	102,880	31,193	30.3	C22:6 *n*-3 (DHA)	269,396	185,991	69.0	C22:6 *n*-3 (DHA)	142,982	46,858	32.6
C20:5 *n*-3 (EPA)	137,695	49,958	36.3	C20:5 *n*-3 (EPA)	58,620	43,965	75.0	C20:5 *n*-3 (EPA)	197,368	61,695	31.0
C20:3 *n*-3	1234	0	0	C20:3 *n*-3	1939	0	0	C20:3 *n*-3	20,833	0	0
				C18:3 *n*-3	1939	4669	Ν/A				N/A
**TOTAL (*n*-3)**	**241,809**	**81,151**	**33.5**	**TOTAL** **(*n*-3)**	**329,955**	**229,956**	**69.0**	**TOTAL (*n*-3)**	**361,183**	**108,553**	**30.0**
**Fish Oil Supplement, Brand D**				**Fish**				**Egg**			
**PUFA**	**C_a_ (mg/kg Lipid)**	**C_b_ (mg/kg** **Lipid)**	**BI** **(%)**	**PUFA**	**C_a_ (mg/kg** **Lipid)**	**C_b_ (mg/kg** **Lipid)**	**BI** **(%)**	**PUFA**	**C_a_ (mg/kg** **Lipid)**	**C_b_ (mg/kg** **Lipid)**	**BI** **(%)**
C22:5 *n*-3 (DPA)	3089	708	22.9	C22:6 *n*-3 (DHA)	389	93	23.9	C22:6 *n*-3 (DHA)	5102	0	0
C21:5 *n*-3	1242	175	14.1	C20:5 *n*-3 (EPA)	254	106	41.7	C22:5 *n*-3	100	0	0
C20:5 *n*-3 (EPA)	24,991	3817	15.3	C18:3 *n*-3	254	40	15.7	C21:5 *n*-3	171	0	0
C20:4 *n*-3	2257	296	13.1	C18:4 *n*-3	0	240	N/A	C18:4 *n*-3	211	0	0
C20:3 *n*-3	506	71	14.4					C18:3 *n*-3	21	579	Ν/A
C18:4 *n*-3	42,269	4523	10.7								
C18:3 *n*-3	0	669	N/A								
**TOTAL (*n*-3)**	**74,354**	**9590**	**12.9**	**TOTAL** **(*n*-3)**	**897**	**239**	**26.6**	**TOTAL (*n*-3)**	**5605**	**0**	**0**

All values are expressed as mean values from at least 2 independent experiments. The BI was calculated for the PUFAs that were initially present in the sample (C_a_ > 0).

**Table 4:** Initial oxidation rates of PUFAs during the digestion of supplement Brand D samples.

Fatty Acid Concentration			
Fatty Acid	C_a_ (ppm)	C_75_ (ppm)	Rate
C18:4 *n*-3	42,269	14,693	−368
C20:5 *n*-3 (EPA)	24,991	7700	−231
C22:5 *n*-3 (DPA)	3089	1412	−22
C20:4 *n*-3	2257	779	−20
C21:5 *n*-3	1242	379	−12
C20:3 *n*-3	506	118	−5
r = −0.9993			

Average rate was calculated as (C_75_ − C_a_)/Δt, where C_75_ is the concentration at 75 min, C_a_ is the initial concentration, and Δt = 75 min (corresponding to the straight line portion of the Figure 3a,b).

## Data Availability

Data available upon request to corresponding author: pkvareltzis@cheng.auth.gr.

## Supplementary Materials

The following are available online, Table S1: List of FA detected via GC-FID along with their nomenclature; Table S2: Concentrations of FA detected via GC-FID.

## Sample Availability

Samples of the raw materials and compounds are available from the corresponding author.
