# Supplementary material for: Bioaccessibility and Oxidative Stability of Omega-3 Fatty Acids in Supplements, Sardines and Enriched Eggs Studied Using a Static In Vitro Gastrointestinal Model
Source: Molecules. 2022 Jan 9;27(2):415. doi: 10.3390/molecules27020415 (PMC8780033; doi:10.3390/molecules27020415)
Supplement: Supplementary file 1 [file molecules-27-00415-s001.zip › molecules-1511264-supplementary.pdf]

molecules

SUPPLEMENTARY MATERIALS

Bioaccessibility and Oxidative Stability of Omega-3 Fatty Acids in Supplements, Sardines and Enriched Eggs Studied Using a Static In Vitro Gastrointestinal Model

Stylianos Floros [1], Alexandros Toskas [2], Evagelia Pasidi [1] and Patroklos Vareltzis [1,*]

[1] Department of Chemical Engineering, Faculty of Engineering, Aristotle University of Thessaloniki, 54124 Thessaloniki, Greece; florstyl@cheng.auth.gr (S.F.); egpasidi@cheng.auth.gr (E.P.)
[2] Petros Androulakis Medical Biology Analytical Laboratories, 57001 Thermi, Greece; alex_tsks@hotmail.com
* Correspondence: pkvareltzis@cheng.auth.gr; Tel: +30-231-099-6162

Table S1. List of FA detected via GC-FID along with their nomenclature.

PUFA	Nomenclature	MUFA	Nomenclature	SFA	Nomenclature
C22:6 n-3 (DHA)	Docosahexaenoic acid	C24:1	Nervonic acid	C24:0	Lignoceric acid
C22:5 n-3 (DPA)	Docosapentaenoic acid	C20:1	Paullinic acid	C21:0	Heneicosylic acid
C21:5 n-3	Heneicosapentaenoate	C20:1 n-9	Gondoic acid	C18:0	Stearic acid
C20:5 n-3 (EPA)	Eicosapentaenoic acid	C18:1 trans	Elaidic acid	C17:0	Margaric acid
C20:4 n-3	Eicosatetraenoic acid	C18:1 cis n-9	Oleic acid	C16:0	Sat. palmitic acid
C20:4 n-6	Arachidonic acid	C18:1 n-7	Vaccenic acid	C15:0	Pentadecylic acid
C20:3 n-3	A-linoleic acid	C17:1	Heptadecenoic acid	C14:0	Myristic acid
C20:3 n-6	Di-homo-γ-linoleic acid	C16:1 n-7	Palmitoleic acid	C13:0	Tridecylic acid
C20:2	Eicosadenoic acid	C15:1	Pentadecenoic acid	C12:0	Lauric acid
C18:4 n-3	Stearidonic acid	C14:1	7-Tetradecenoic acid	C11:0	Undecylic acid
C18:3 n-3	α-linolenic acid			C10:0	Capric acid
C18:3 n-6	γ-linolenic acid			C8:0	Caprylic acid
C18:2 cis	Linoleic acid			C6:0	Caproic acid
C18:2 trans	Linolelaidic acid			C4:0	Butyric acid
C18:2 cis	Octadecadienoic acid				

Table S2. Concentrations of FA detected via GC-FID.

Sample	PUFA	Raw State	Digested State	MUFA	Raw State	Digested State	SFA	Raw State	Digested State
**B**	C22:6 n-3	102,880	31,193	C20:1	3,868	0	C18:0	16,707	7,407
**R**	C20:5 n-3	137,695	49,958	C18:1 cis n-9	1,893	740	C17:0	576	0
**A**	C20:4 n-6	17,283	5,432	C18:1 trans	740	713	C16:0	22,304	15,363
**N**	C20:3 n-3	1,234	0	C17:1	1,481	932	C15:0	411	0
**D**	C20:3 n-6	17,283	5,432	C16:1	576	4,004	C14:0	5,102	4,471
	C20:2	1,810	0				C10:0	1,316	383
**A**	C18:3 n-6	2,798	3,621				C8:0	1,728	416
	C18:2 cis	7,407	3,210						
	**TOTAL**	**288,390**	**98,846**		**8,558**	**6,389**		**48,144**	**28,040**
**B**	C22:6 n-3	269,396	185,991	C20:1	1,508	0	C18:0	49,568	33,692
**R**	C20:5 n-3	58,620	43,965	C18:1 cis n-9	84,913	58,835	C17:0	1,724	1,005
**A**	C20:4 n-6	22,198	15,876	C16:1	862	0	C14:0	6,465	3,807
**N**	C20:3 n-6	2,586	0				C12:0	1,939	1,149
**D**	C20:3 n-3	1,939	0				C10:0	34,482	21,838
	C20:2	3,017	1,436				C8:0	50,646	30,028
**B**	C18:3 n-3	1,939	4,669				C6:0	3,879	2,586
	C18:3 n-6	6,250	3,520						
	C18:2 trans	3,017	0						
	C18:2 cis	14,439	12,643						
	**TOTAL**	**383,401**	**268,100**		**87,283**	**58,835**		**148,703**	**94,105**
**B**	C22:6 n-3	142,982	46,858	C18:1 cis n-9	65,570	98,537	C18:0	29,824	22,952
**R**	C20:5 n-3	197,368	61,695	C18:1 trans	1,096	4,020	C17:0	2,850	0
**A**	C20:4 n-6	1,535	8,918	C17:1	1,973	4,531	C16:0	35,964	71,927
**N**	C20:3 n-3	20,833	0	C16:1	17,324	15,423	C14:0	7,894	22,806
**D**	C20:3 n-6	2,412	0				C10:0	1,535	0
	C20:2	2,192	0				C8:0	1,754	0
**C**	C18:3 n-6	3,508	24,123						
	C18:2 cis	10,087	149,122						
	**TOTAL**	**380,917**	**290,716**		**85,963**	**122,511**		**79,821**	**117,685**
**B**	C22:5 n-3	3,089	708	C20:1 n-9	5,506	779	C21:0	177	0
**R**	C21:5 n-3	1,242	175	C20:1	2,232	283	C18:0.	4,000	583
**A**	C20:5 n-3	24,991	3,817	C18:1 cis n-9	11,633	5,629	C17:0	119	0
**N**	C20:4 n-3	2,257	296	C18:1 n-7	54,198	1,559	C16:0	29,069	3,697
**D**	C20:4 n-6	26,485	3,221	C18:1 trans	182	87	C15:0	935	124
	C20:3 n-3	506	71	C17:1	1,511	290	C14:0	12,648	1,841
**D**	C20:3 n-6	288	0	C16:1 n-7	26,997	3,552	C12:0	100	0
	C20:2	760	136	C14:1	361	44	C10:0	355	46
	C18:4 n-3	42,269	4,523				C8:0	468	49
	C18:3 n-6	351	66						
	C18:3 n-3	0	669						
	C18:2 n-4 cis	4,569	631						
	C18:2 n-6	828	124						
	C18:2 trans	1,188	0						
	**TOTAL**	**108,823**	**14,437**		**102,620**	**12,223**		**47,871**	**6,340**
**F**	C22:6 n-3	389	93	C20:1 cis n-9	50	160	C18:0	186	3,840
**I**	C20:5 n-3	254	106	C18:1 cis n-9	68	7,960	C17:0	34	53

**S**	C20:4 n-6	0	66	C18:1 trans	1,881	786	C16:0	728	12,493
**H**	C20:3 n-6	0	40	C18:1 n-7	745	213	C15:0	33	466
	C18:4 n-3	0	240	C17:1	0	93	C14:0	186	4,320
	C18:3 n-6	0	40	C16:1 n-7	118	253	C12:0	0	1,346
	C18:3 n-3	254	40	C14:1	0	386	C10:0	0	1,173
	C18:2 n-6	0	80				C8:0	0	440
	C18:2 n-4 cis	0	1,013				C6:0	0	640
	C18:2 trans	203	26				C4:0	0	813
	**TOTAL**	**1,100**	**1,744**		**2,862**	**9,851**		**1,167**	**25,584**
**E**	C22:6 n-3	5,102	0	C20:1	0	85	C22:0	46	0
**G**	C22:5 n-3	100	0	C18:1 n-7	1,940	378	C18:0	7,980	855
**G**	C21:5 n-3	171	0	C18:1 cis n-9	40,715	8,833	C17:0	184	0
	C20:4 n-6	1,715	2,013	C18:1 trans	151	0	C16:0	23,241	2,802
	C20:3 n-6	0	143	C17:1	124	679	C14:0	22,204	13,761
	C20:2	82	0	C16:1 n-7	2,497	593	C4:0	0	4,170
	C18:4 n-3	211	0	C14:1	54	0			
	C18:3 n-3	21	579						
	C18:3 n-6	0	60						
	C18:2 cis n-4	16,863	3,613						
	C18:2 trans	6,838	0						
	**TOTAL**	**31,103**	**6,408**		**45,481**	**10,568**		**53,655**	**21,588**
